# tRFUniverse: A comprehensive resource for the interactive analyses of tRNA-derived ncRNAs in human cancer

**DOI:** 10.1016/j.isci.2024.108810

**Published:** 2024-01-05

**Authors:** Alessandro La Ferlita, Salvatore Alaimo, Giovanni Nigita, Rosario Distefano, Joal D. Beane, Philip N. Tsichlis, Alfredo Ferro, Carlo M. Croce, Alfredo Pulvirenti

**Affiliations:** 1 Department of Cancer Biology and Genetics, The James Comprehensive Cancer Center, The Ohio State University, Columbus, OH, USA; 2 Department of Clinical and Experimental Medicine, Knowmics Lab, University of Catania, Catania, Italy; 3 Department of Surgery, Division of Surgical Oncology, The James Comprehensive Cancer Center, The Ohio State University, Columbus, OH, USA

**Keywords:** Nucleic acids, Bioinformatics, Cancer, Transcriptomics

## Abstract

tRNA-derived ncRNAs are a heterogeneous class of non-coding RNAs recently proposed to be active regulators of gene expression and be involved in many diseases, including cancer. Consequently, several online resources on tRNA-derived ncRNAs have been released. Although interesting, such resources present only basic features and do not adequately exploit the wealth of knowledge available about tRNA-derived ncRNAs. Therefore, we introduce *tRFUniverse*, a novel online resource for the analysis of tRNA-derived ncRNAs in human cancer. *tRFUniverse* presents an extensive collection of classes of tRNA-derived ncRNAs analyzed across all the TCGA and TARGET tumor cohorts, NCI-60 cell lines, and biological fluids. Moreover, public AGO CLASH/CLIP-Seq data were analyzed to identify the molecular interactions between tRNA-derived ncRNAs and other transcripts. Importantly, *tRFUniverse* combines in a single resource a comprehensive set of features that we believe may be helpful to investigate the involvement of tRNA-derived ncRNAs in cancer biology.

## Highlights

• TRNA-derived ncRNAs are active regulators of gene expression involved in cancer
• TRFUniverse is a novel online app for analyzing tRNA-derived ncRNAs in cancer
• Analyses available in TCGA/TARGET cohorts, NCI60 cell lines, and biological fluids
• Targets of tRNA-derived ncRNAs identified in public AGO CLASH/CLIP-Seq data

## Graphical abstract

**Figure:**
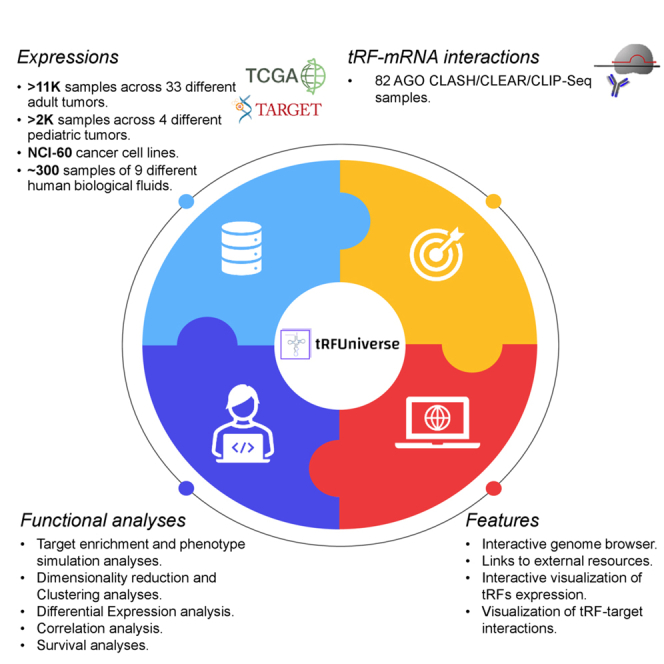

## Introduction

With the advent of Next Generation Sequencing (NGS) technologies, the number of identified non-coding RNAs (ncRNAs) classes in eukaryotic cells has dramatically increased.^1^^,^^2^^,^^3^ Recently, tRNA-derived ncRNAs, a heterogeneous group of ncRNAs originating from tRNA processing, have been characterized.^4^^,^^5^^,^^6^ tRNA biogenesis begins with the transcription of tRNA genes by RNA polymerase III, leading to precursor tRNA (pre-tRNA). Such molecules undergo a maturation process inside the nucleus where 5′ leader and 3′ trailer sequences are cleaved by ribonuclease P (RNase P) and ribonuclease Z (RNase Z), respectively.^7^^,^^8^^,^^9^^,^^10^^,^^11^^,^^12^^,^^13^^,^^14^ Several kinds of tRNA-derived ncRNAs have been discovered in the last few years.^14^^,^^15^ However, a universally accepted nomenclature is still missing. A typical grouping of such molecules is based on the location of cleavage sites within the tRNA. tRNA-derived ncRNAs can therefore be divided in: (i) tRNA-derived fragments (tRFs), which derive from the cleavage of either the D- or T-loop of the mature tRNA (tRF-5 and tRF-3, respectively)^6^^,^^11^; (ii) tRNA-halves which are generated by specific cleavage in the anticodon region of the mature tRNA^16^ (tRNA-halves can also be produced by an RNAse operated cleavage in the anticodon region that occurs under stress condition and, therefore, named stress-induced tRNA fragments (tiRNAs).^17^ Note, the term tiRNA was first coined by^18^); (iii) tRNA-derived small RNAs (tsRNAs), which derive from the 3′ trailer sequence of the pre-tRNA^19^ (also named tRF-111); and (iv) 5′ leader tRF which are generated from the 5′ leader sequence of the pre-tRNA.^20^ More details about tRNA-derived ncRNA classification and nomenclature can be found in.^21^^,^^22^ Importantly, it has been shown that tRNA-derived ncRNAs are not mere byproducts of random tRNA cleavage as reported in.^11^^,^^23^ Rather they may actively play roles in several biological phenomena, such as ribosome biogenesis, retrotransposition, virus infections, apoptosis, and cancer pathogenesis.^6^^,^^19^^,^^24^^,^^25^^,^^26^^,^^27^^,^^28^^,^^29^^,^^30^^,^^31^^,^^32^^,^^33^ Furthermore, some tRNA-derived ncRNA classes have been shown to bind PIWI and AGO proteins, potentially acting as pre- or post-transcriptional regulators of gene expression, respectively.^19^^,^^34^ Accumulating evidence also indicates the presence of functional tRNA-derived ncRNAs in human biological fluids, such as urine and serum, from patients with cancer.^7^^,^^35^^,^^36^^,^^37^^,^^38^^,^^39^

Because of the scientific community’s increasing interest in studying the roles of tRNA-derived ncRNAs in cancer biology, several online resources have been released in the last few years. The first resource was a database released in 2014 by Kumar P. et al., named *tRFdb*.^40^ After that, other databases were released, such as *tRF2Cancer*^41^ and *MINTbase*.^42^ These databases were just catalogs of tRNA-derived ncRNAs expressed in several tumor types. Therefore, they gave users the opportunity to navigate among these RNA molecules and their features but they did not give them the tools to perform functional analyses.

To address the problem, we released in 2019 *tRFexplorer*,^21^ the first online resource that allowed users to search for tRNA-derived ncRNAs and visualize their expression profiles in NCI-60 cell lines and The Cancer Genome Atlas (TCGA) patient cohorts.^43^ Moreover, through *tRFexplorer*, users could perform differential expression analyses on TCGA samples, correlating tRNA-derived ncRNA expression with several covariates, such as NCI-60 expression data and drug sensitivity, and mRNA/miRNA expression across the TCGA cohorts.^21^ Since then, several newer online resources on tRNA-derived ncRNAs in cancer have been released. Noteworthy examples include *OncotRF*,^44^ *tsRBase*,^45^ *tRFtarget*,^46^ tRFTar,^47^ *tRFTars*,^48^ and *tsRFun*^49^ (which is the upgrade of *tRF2cancer*^41^). These resources presented some interesting features, such as reporting molecular interactions between tRNA-derived ncRNAs and mRNAs, and performing target enrichment, differential expression, and survival analyses. However, such web applications continue to exhibit limitations: (i) there are missing tRNA-derived ncRNA classes; (ii) the functional analysis tools they provide are limited; and (iii) they fail to provide a single integrated portal that includes most of the features required for a comprehensive analysis.

To address the preceding issues, we developed *tRFUniverse* (https://trfuniverse.cloud/), a novel web application for the interactive analysis of tRNA-derived ncRNAs in human cancer. *tRFUniverse* presents the most extensive collection of different classes of tRNA-derived ncRNAs analyzed across all the small RNA sequencing (smRNA-Seq) data available for the TCGA cohorts, NCI-60 cell lines, human biological fluids, and, for the first time, also across all the pediatric tumors available on TARGET. Moreover, several AGO CLASH/CLEAR/CLIP-Seq data have been analyzed to identify the molecular interactions between tRNA-derived ncRNAs and other transcripts. Analysis of such data is allowed to users through a web-based and easy-to-use Graphical User Interface (GUI), that consents (i) to explore the expression of tRNA-derived ncRNAs across all TCGA, TARGET, NCI-60, and biological fluids data; (ii) to perform differential expression analyses on the TCGA and TARGET patient cohorts; (iii) to perform correlation analyses between tRNA-derived ncRNAs and mRNA\miRNA expression in TCGA and TARGET samples; (iv) to navigate through the tRNA-derived ncRNAs-mRNA interactions identified in the AGO CLASH/CLEAR/CLIP-Seq data; (v) to perform pathway enrichment analysis with genes which are either correlated or targeted by tRNA-derived ncRNAs; (vi) to perform survival analyses based on tRNA-derived ncRNA expression in TCGA and TARGET samples as well as many others functional analyses aiming to help researchers to investigate the roles of these small RNA molecules in cancer biology.

## Results

### Data included in tRFUniverse

*tRFUniverse* is a publicly available online resource developed to provide users with an easy-to-use online resource capable of: (i) enabling the interactive exploration of tRNA-derived ncRNA expression in many different types of human cancer; (ii) and performing several analyses helpful to investigate the biological functions of these small RNA molecules in each analyzed tumor.

In order to achieve our goal, we first analyzed the expression of the tRNA-derived ncRNAs in all the smRNA-Seq data available in TCGA^43^ and TARGET (http://ocg.cancer.gov/programs/target), which accounts for more than 13000 samples spanning across 33 and 4 different tumor types for adult and pediatric tumors, respectively. The number of samples analyzed for each TCGA\TARGET cohort is reported in Table S1. In addition to the TCGA\TARGET samples, we (as in the first version of our resource) provide information about tRNA-derived ncRNA expression in commonly used human cancer cell lines. For this reason, we also analyzed the publicly available smRNA-Seq data of the NCI-60 cell lines, a panel of 60 distinct cancer cell lines spanning nine different types of human tumors (Table S2). As a result, we identified 44,456 tRNA-derived ncRNAs expressed in TCGA\TARGET cohorts and NCI60 cell lines, and, therefore, we included them in *tRFUniverse*. The numbers of tRNA-derived ncRNAs (for each subclass) expressed in the analyzed datasets are reported in Table S3. Moreover, to implement in *tRFUniverse* the functional analyses described in the next section, clinical information together with gene and microRNA (miRNAs) expression matrices (raw counts) for all the TCGA\TARGET and NCI-60 samples were downloaded from the GDC data portal.

**Figure 1:**
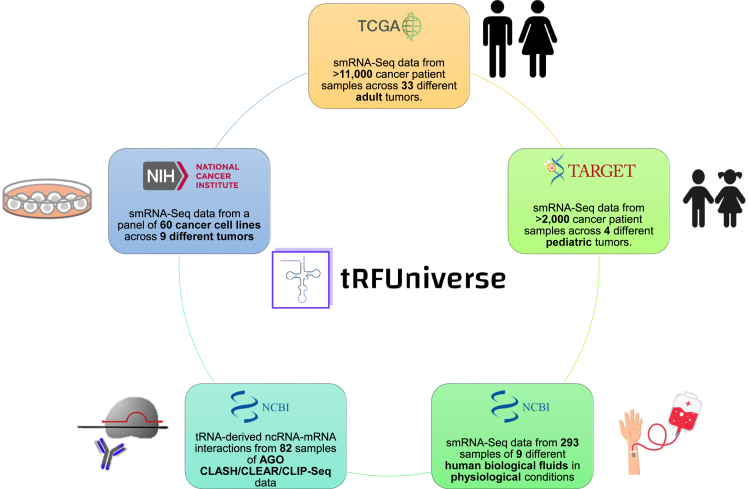
Analyzed data Schematic representation of the data that has been analyzed for tRFUniverse.

The core of *tRFUniverse* consists of the tRNA-derived ncRNAs expressed in TCGA\TARGET and NCI-60. To extend the current knowledge about such molecules, we also analyzed smRNA-Seq data retrieved from several human biological fluids and AGO CLASH/CLEAR/CLIP-Seq data. This allowed us to both establish their presence in human biological fluids and identify their molecular target. In more detail, 293 samples from several human fluids in physiological conditions, such as seminal fluid, saliva, serum, urine, plasma, stool, ovarian follicular fluid, cerebrospinal fluid, and bile, were analyzed. Concerning the AGO CLASH/CLEAR/CLIP-Seq data, 82 samples were analyzed, providing direct evidence of tRNA-derived ncRNAs-mRNAs interactions in several different biological samples. The lists of analyzed publicly available smRNA-Seq data of biological fluids and AGO CLASH/CLEAR/CLIP-Seq data are reported in Tables S4 and S5, respectively. A schematic representation of the *tRFUniverse* data is shown in Figure 1.

### Features available within tRFUniverse

**Figure 2:**
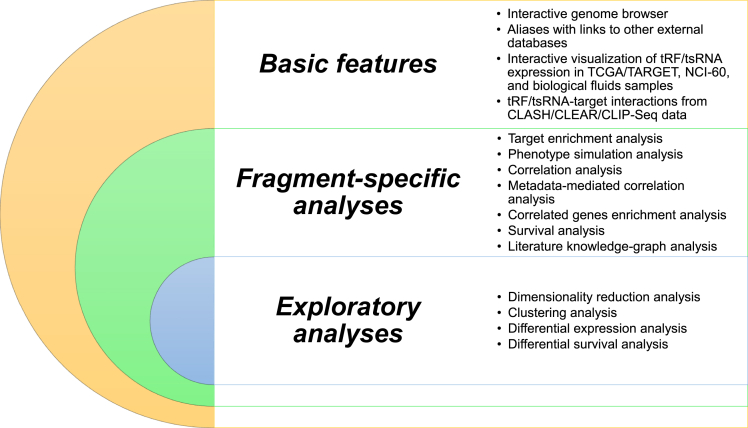
Features and analyses Classification of all the features and analyses implemented in tRFUniverse.

*tRFUniverse* presents several features, and analysis modules developed to provide researchers with information that might help investigate the role of tRNA-derived ncRNAs in cancer biology (Figure 2). Before describing all the functional analyses implemented in *tRFUniverse*, we first briefly report the crucial features in exploring the tRNA-derived ncRNAs reported in our web application. *tRFUniverse* presents a very intuitive GUI that facilitates the exploration of all the tRNA-derived ncRNAs expressed in the TCGA\TARGET cohorts and NCI-60 cell lines. Users can filter the tRNA-derived ncRNAs from the “search” tab based on their average expression in a specific cohort, the fragment type, the chromosome where they are transcribed, and the anticodon and amino acid of the tRNA from where they are processed. In addition, all the tRNA-derived ncRNAs available in *tRFUniverse* can be explored from the “Browse” tab and filtered accordingly with their name, sequence length, and type. Either way, by selecting a specific tRNA-derived ncRNA, it is possible to navigate among its information using the several features implemented in *tRFUniverse* as described later in discussion.

#### Genome browser

An interactive genome browser is available to visualize both the genomic location where that specific tRNA-derived ncRNA is transcribed together with its sequence. In addition, information such as chromosome, coordinates, strand, amino acid, and anticodon of the tRNA of origin are reported.

#### Alias

A unique nomenclature for tRNA-derived ncRNAs is still missing. Commonly used IDs for tRNA-derived ncRNAs are reported on tRFdb^40^ and MINTbase.^42^ However, other online databases assign different IDs to these short RNA molecules.^44^^,^^45^^,^^49^ To avoid confusion, *tRFUniverse* reports all the aliases of each tRNA-derived ncRNA, linking the external resources.^44^^,^^45^^,^^49^

#### Expression

For each tRNA-derived ncRNA available in *tRFUniverse*, it is possible to visualize its expression in all the TCGA\TARGET cohorts, NCI-60 cell lines, and biological fluids. This plot can be generated by selecting (i) a cohort/cell line/fluid; (ii) the associated metadata (e.g., sex, stage, survival, type of sample, histological subtypes, molecular subtype); and (iii) how the expression should be presented among some options such as raw counts, Read Per Million (RPM), or normalized counts (with Limma’s voom function). Normalized counts can also be corrected for batch effect by selecting among several covariates, such as sex, age, and tumor purity. This latter was calculated as in.^50^ The expression is finally shown as an interactive boxplot that can also be downloaded for the user’s convenience.

#### Targets

A list of mRNA targets is reported for each tRNA-derived ncRNA available in *tRFUniverse*. Indeed, as it is known from the literature, tRNA-derived ncRNAs, among several other functions, are known to be complexed with the AGO proteins acting, therefore, as post-transcriptional regulators with a miRNA-like manner.^34^^,^^51^^,^^52^ For this reason, we decided to analyze publicly available AGO CLASH/CLEAR/CLIP-Seq data with the scope to identify the molecular targets of the tRNA-derived ncRNAs identified as expressed in TCGA\TARGET cohorts and NCI-60 cell lines. The analysis of such data revealed that not only the tRFs generated from the mature tRNA were incorporated in the AGO complex but also tsRNAs and 5′ leader tRFs were detected. For each tRNA-derived ncRNA, targets are reported in an interactive table that can be filtered by searching for a specific gene using either the gene symbol or ENSEMBL ID and sorted using the number of binding sites present in that specific gene or the Minimum Free Energy (MFE). By selecting a specific target, it is also possible to visualize the transcript IDs, the position of the binding sites (5′ UTR, CDS, 3′ UTR), the number of evidence, the start and end of the binding site, and the MFE. Moreover, information about the dataset, type of experimental data (CLASH, CLEAR, or CLIP), AGO protein, and sequence of that specific interaction is also reported.

### Analyses of tRFUniverse

In addition to the basic features described in the previous section, several functional analyses (Figure 2) have been implemented to provide users with a tool that might help them investigate tRNA-derived ncRNAs in several human cancers. With this aim, we implemented two major classes of analyses: (i) fragment-specific analyses and (ii) exploratory analyses (Figure 2). The first class of analyses is oriented toward investigating the potential biological functions of a specific tRNA-derived ncRNA (also called “fragment”) in the context of specific or multiple tumor types. In contrast, the second class of analyses is tumor-centric. It aims to identify all the tRNA-derived ncRNAs potentially involved in the development or progression of that tumor type.

Concerning the fragment-specific analyses, these include (i) target enrichment analysis; (ii) phenotype simulation analysis; (iii) correlation analysis; (iv) metadata-mediated correlation analysis; (v) correlated gene enrichment analysis; (vi) survival analysis; and (vii) literature knowledge-graph analysis. These analyses can be run after selecting a specific fragment using either the “search” or “browse” tab from the homepage of *tRFUniverse* and clicking on the desired analysis as described later in discussion.

#### Target enrichment analysis

Using the targets identified in the AGO CLASH/CLEAR/CLIP-Seq data, it is possible to perform target enrichment analysis to identify which pathways are enriched when selecting a specific tRNA-derived ncRNA. This analysis may indicate which biological pathways might be regulated by that specific tRNA-derived ncRNA. To run the analysis, users have to select which type of data (CLASH, CLEAR, CLIP, or a combination of them) must be used to get the targets of that specific tRNA-derived ncRNA. Moreover, users can optionally filter the potential targets by selecting only those “active” in a specific dataset. In this case, for active targets, we mean genes potentially regulated by that specific tRNA-derived ncRNA. The activity is calculated by performing a differential expression analysis between samples with high fragment expression (>75 percentile) and samples with low fragment expression (<25 percentile). Then, a p value threshold (defined by the user) is applied to the differential expression analysis results. All targets with a p value below the threshold are considered active in the dataset. This optional filtering is important to select only those targets expressed and potentially regulated in a specific TCGA\TARGET cohort. The results are finally presented as an interactive table that lists all the enriched pathways, with their IDs, descriptions, target genes, and the number of targeted genes for each pathway. The results can also be downloaded as a CSV, Excel, or JSON file.

#### Phenotype simulation analysis

In addition to the target enrichment analysis, a more complex way to investigate which pathways may be regulated by the user-selected tRNA-derived ncRNA is through a phenotype simulation analysis. In more detail, in *tRFUniverse,* we have included PHENSIM,^53^^,^^54^ a phenotype simulator that relies on the target interactions identified from the AGO CLASH/CLEAR/CLIP-Seq data and the topological information encoded on the KEGG^55^^,^^56^^,^^57^ and REACTOME^58^^,^^59^^,^^60^^,^^61^ pathways to identify the metabolic and signaling pathways potentially regulated by a specific tRNA-derived ncRNA and, in turn, its effects on the cellular phenotype. This is important since both tRFs generated from the mature tRNA^34^^,^^51^ and the 5′ leader and 3′ trailer tRFs generated from the precursor of the tRNA^19^ have been shown to be bound to AGO proteins, functioning as active regulators of gene expression. However, users should consider that these simulations are limited by data availability. Indeed, since different tRNA-derived ncRNAs are produced in different tissues,^62^ and we have a limited set of AGO CLASH/CLEAR/CLIP-Seq data in limited experimental conditions, some important biological targets might be missing from the data. However, this is not an inherent limitation of the simulation model that can be expanded as soon as more data are available.

To perform such analysis, as in the previous case, users have to select which type of data (CLASH, CLEAR, CLIP, or a combination of them) has to be used to get the targets of that specific tRNA-derived ncRNA, and optionally, filter the targets by selecting only those that are potentially regulating those targets in a specific dataset as previously described for the target enrichment analysis. Once the analysis is completed, the results are shown in an interactive table that reports all the KEGG and REACTOME pathways found to be regulated by the user-selected tRNA-derived ncRNA with their IDs, activity scores, p values and false discovery rate (FDR). By clicking on a specific pathway, another interactive table is shown, listing all the potentially affected genes included in that pathway. All the results tables can also be downloaded as a CSV, Excel, or JSON file.

#### Correlation analysis

Looking at the potential direct targets of a specific tRNA-derived ncRNA is not the only way to infer its biological function. Indeed, tRNA-derived ncRNAs may regulate gene expression with various mechanisms, mostly unknown, that go beyond the simple direct targeting of specific mRNAs. Therefore, a more agnostic approach, like a simple correlation analysis, may highlight potentially relevant direct or indirect connections between tRNA-derived ncRNA and gene expression that otherwise would not be possible to detect. For this reason, in *tRFUniverse*, we allow users to perform correlation analyses between tRNA-derived ncRNA and gene\miRNA expression. For performing such analysis, users have to select (i) a TCGA\TARGET cohort or NCI-60 cell line; (ii) the correlation measure (Pearson, Spearman, or Kendall); and (iii) if genes or miRNAs must be correlated with the expression of the selected tRNA-derived ncRNA. In addition to these required parameters, others are optional. These include (i) covariates for batch correction (age, sex, sample type, race, tumor mutational burden, subtypes, tumor purity, and others); (ii) samples’ histological or molecular subtypes filter; and (iii) samples’ type filter. The results are presented as an interactive table that shows all the correlated genes using their gene symbols and ENSEMBL IDs, the correlation coefficient value, p value, and adjusted p value. Moreover, a downloadable and interactive scatterplot is also shown by clicking on a specific correlated gene. The full results table can also be downloaded as a CSV, Excel, or JSON file.

#### Metadata-mediated correlation analysis

Even though correlation analyses might help identify direct or indirect connections between tRNA-derived ncRNA expression and regulation of specific genes, the considerable diversity of tumor samples, even within the same molecular or histological subtype, sometimes makes it hard to identify such connections. For this reason, on some occasions, it is essential to look at correlations, considering at the same time additional clinical or molecular variables about the selected subset of samples.^63^ Such information includes sex, sample type, sample subtype, survival status, potential treatments, race, tumor purity, tumor mutational burden, and so on. Such analysis may identify correlations that otherwise may not be statistically significant.^63^ For this reason, in *tRFUniverse,* we have implemented the opportunity to perform metadata-mediated correlation analysis. To run the analysis, users must select (i) a TCGA\TARGET cohort or NCI-60 cell line; (ii) the metadata to be used for the correlation correction; and (iii) if genes or miRNAs must be correlated with the expression of the selected tRNA-derived ncRNA. In addition to these mandatory parameters, users might also select (i) the covariates for batch correction (age, sex, sample type, race, tumor mutational burden, subtypes, tumor purity, and others); (ii) the samples’ histological or molecular subtypes to be used for the analysis; and also (iii) the samples’ type. The results of such analysis are reported as an interactive table that includes the list of correlated genes with their symbols and ENSEMBL IDs, p values, and adjusted p values. The table can also be sorted using the previously mentioned information and downloaded as a CSV, Excel, or JSON file. Moreover, by clicking on a specific correlated gene, an interactive and downloadable scatterplot showing the correlation between the selected tRNA-derived ncRNA and the gene of interest is also shown.

#### Correlated gene enrichment analysis

As we previously said, identifying correlated genes is another way to look at the possible biological functions of a specific tRNA-derived ncRNA. In fact, the expression of correlated genes may be directly or indirectly affected by the expression of tRNA-derived ncRNAs. At that point, it may be helpful to know which biological pathways are enriched when selecting all the genes correlated with a specific tRNA-derived ncRNA. For this reason, in *tRFUniverse*, we allow users to perform pathway enrichment analysis using as input the list of genes correlated with the selected tRNA-derived ncRNA. In more detail, users must select (i) the dataset to be analyzed; (ii) the correlation measure (Pearson, Spearman, or Kendall); (iii) the minimum absolute correlation threshold; and (iv) if genes or miRNAs must be correlated with the expression of the selected tRNA-derived ncRNA. In addition to these mandatory parameters, others are optional. These include (i) covariates for batch correction (age, sex, sample type, race, tumor mutational burden, subtypes, tumor purity, and others); (ii) filter for samples’ histological or molecular subtypes; and (iii) filter for samples’ type. After running the analysis, the results are reported as an interactive table that lists all the enriched pathways with their IDs and descriptions, the correlated genes included in each enriched pathway, the gene ratio, p value, and adjusted p value. As for all the analyses implemented in *tRFUniverse*, the results table can be downloaded as a CSV, Excel, or JSON file.

#### Survival analysis

Another crucial biological aspect to look at in a specific tRNA-derived ncRNA is its potential association with the differences in survival in a given tumor type. Indeed, this may indicate if that fragment may be associated with the development and progression of a specific tumor. For this reason, in *tRFUniverse*, it is possible to perform survival analyses in all TCGA and TARGET cohorts after selecting a tRNA-derived ncRNA. In more detail, to run the analysis, users must select (i) the dataset (TCGA\TARGET cohorts); (ii) the survival measure (overall survival, disease-free survival, disease-specific survival, or progression-free survival); (iii) the used expression values (counts, RPM, or normalized counts); and (iv) the cutoff to define a sample with a low or high fragment’s expression (median, quartile, or custom). In addition to these required parameters, others are optional. These optional parameters allow users to filter the samples to be analyzed based on their (i) histological or molecular subtypes; (ii) types (e.g., primary tumor, metastasis); and (iii) survival range. The results are shown as an interactive and downloadable Kaplan-Meier curve showing the statistical significance (p value) and Hazard Ratio (HR) value.

#### Literature knowledge-graph analysis

As a final fragment-specific analysis, in *tRFUniverse*, we allow users to build a knowledge graph based on the information available about the selected tRNA-derived ncRNA from the literature. Such a function is powered by NetME,^64^^,^^65^ a novel text-mining software that, starting from a set of full texts obtained from PubMed, can extract biological elements from ontological databases and then synthesize a network inferring relations among such elements. In this case, *tRFUniverse* creates a query to NetME using as a biological element the tRNA-derived ncRNA selected from the user with all its potential aliases reported in other databases. At that point, if that tRNA-derived ncRNA has been reported in some research article, NetMe will be able to build the fragment-specific knowledge-based graph that may be used to explore its biological functions or association with certain diseases. However, since many tRNA-derived ncRNAs available in *tRFUniverse* have never been studied and, therefore, never mentioned in the scientific literature, for them, such a knowledge-based graph cannot be generated. More details about NetME can be found on its website (https://netme.click/#/) and publication.^64^

Concerning the exploratory functional analyses, we implemented (i) dimensionality reduction analysis; (ii) clustering analysis; (iii) differential expression analysis; and (iv) differential survival analysis. These analyses can be run by clicking the "Analysis" tab from the homepage and selecting the desired analysis. After that, a specific web page will open to request that the user select all the required parameters to run the analysis correctly. In what follows, we briefly discuss the exploratory functional analyses implemented in *tRFUniverse*.

#### Dimensionality reduction

In *tRFUniverse,* we allow users to perform dimensionality reduction analyses to see if it is possible to identify different clusters of samples within a user’s selected subset in a two-dimensional space using the expression profile of the tRNA-derived ncRNAs. This type of analysis might be helpful to identify signatures of tRNA-derived ncRNAs that might be used to discriminate between different tumor subtypes or even define a new molecular sub-classification of a specific tumor. The analysis can be easily run by selecting (i) the datasets to be analyzed; (ii) the metadata that must be used to annotate the samples; (iii) the dimensionality reduction algorithm (PCA, MDS, ICA, tSNE, and UMAP); (iv) the covariates for batch correction (age, sex, sample type, race, tumor mutational burden, subtypes, tumor purity, and others); (v) the tumor histological or molecular subtypes; (vi) and the sample types. In addition to all the previously mentioned parameters, specific options are displayed according to the selected dimensionality reduction algorithm for selecting the tRNA-derived ncRNAs to be used for the analysis. The analysis results are finally shown as an interactive dot plot that can also be downloaded for the user’s convenience.

#### Clustering

In addition to the previously described dimensionality reduction analysis, in *tRFUniverse,* we have implemented the opportunity to perform clustering analyses that are visualized in an interactive and customizable heatmap. As for the dimensionality reduction analysis, this type of analysis can be run to identify clusters of samples that show similar tRNA-derived ncRNA expression patterns. The analysis can be run by selecting (i) the dataset to be analyzed; (ii) the metadata that should be used for the samples’ annotation; (iii) the covariates for the batch correction; (iv) the tumor subtypes; and (v) samples’ type. In addition to these parameters, users must also define the coefficient (mean absolute deviation, variance, absolute median, and absolute mean) to be used to calculate the variability in tRNA-derived ncRNA expression within the selected dataset and the number of the most variable tRNA-derived ncRNAs that should be used for the clustering analysis. As previously said, the results are shown as an interactive and customizable heatmap that can also be downloaded in various formats (PDF, PNG, and SVG).

#### Differential expression analysis

Another useful analysis implemented in *tRFUniverse* is differential expression analysis. Such analysis is crucial to identify tRNA-derived ncRNAs that have an altered expression in a specific condition and that may be implicated with the development or progression of that malignancy. The differential expression analysis can be run from the “Analysis” tab present on *the tRFUniverse* homepage and selecting all the required parameters. Such parameters include (i) the dataset to be analyzed (TCGA\TARGET cohorts); (ii) the relative metadata; (iii) the covariates for batch correction; (iv) and the contrasts to be performed. In addition to that, users can also define the cutoff for the logFC and q-value for considering a specific tRNA-derived ncRNA as differentially expressed. Moreover, users can also filter lowly expressed tRNA-derived ncRNAs from the analysis by removing all the fragments that have a number of reads under a user’s defined cutoff. The analysis results are reported as an interactive table, downloadable as a CSV file. In addition to the results table, an interactive and downloadable volcano plot is also shown.

#### Differential survival analysis

The last exploratory functional analysis implemented in *tRFUniverse* is the survival analysis. Such analysis allows users to identify all the tRNA-derived ncRNAs associated with statistically significant differences in survival in a specific tumor type. To run the analysis, users have to select (i) the dataset (TCGA\TARGET cohorts); (ii) the survival measure (overall survival, disease-free survival, disease-specific survival, or progression-free survival); (iii) the type of expression value used for the analysis (counts, RPM, or normalized counts); (iv) and the cutoff to define a sample with a low or high fragment’s expression (median, quartile, or custom). In addition to the previously required parameters, users can filter samples from the selected TCGA\TARGET cohort by selecting the tumor histological or molecular subtype and the sample type. Moreover, users can optionally select the time range for the survival analysis. As for the other analyses implemented in *tRFUniverse*, the results are reported as an interactive table that lists all the tRNA-derived ncRNAs that are associated or not with differences in survival with their relative statistics (HR, p value, and q-value). Such a table can also be downloaded as a CSV, Excel, or JSON file. Moreover, by clicking on a specific fragment, an interactive and downloadable Kaplan-Maier curve reporting HR and p value is shown.

### Comparison with other online resources

In recent years, several other resources for tRNA-derived ncRNAs have been released, such as *MINTbase*,^42^ *OncotRF*,^44^ *tRFdb*,^40^ *tRFtarget*,^46^ *tRFTar*,^47^ *tRFTars*,^48^ *tsRBase*,^45^ and *tsRFun*^49^ (which is the upgraded version of *tRF2cancer*^41^). In order to show the potential applicability of *tRFUniverse*, we evaluated our resource with the previously mentioned databases. In more detail, a qualitative evaluation was performed by comparing all the previously mentioned databases in terms of (i) reported classes of tRNA-derived ncRNAs; and (ii) implemented features and analyses.

**Figure 3:**
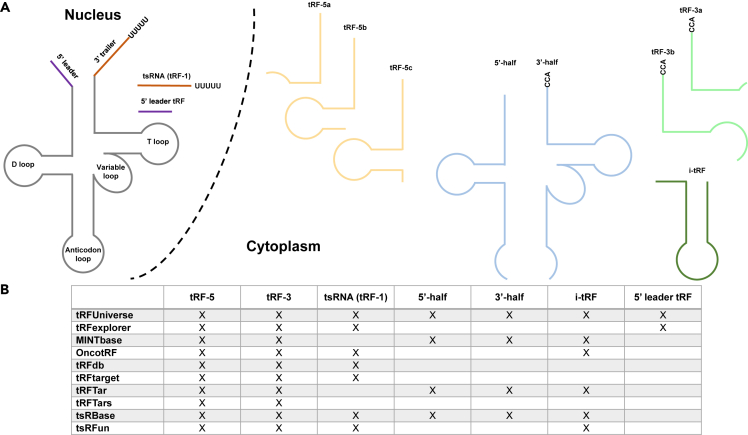
tRNA-derived ncRNA classes and comparison table (A) Schematic representation of all the different classes of tRNA-derived ncRNAs. (B) Table showing all the different classes of tRNA-derived ncRNAs reported in tRFUniverse, and in the other compared databases.

Concerning the tRNA-derived ncRNA classes available in the compared databases, *tRFUniverse* is the only one reporting all the different classes so far discovered (Figure 3A). Indeed, databases such as *MINTbase* and *tRFTar* report only tRNA-derived ncRNAs generated from the processing of mature tRNA (tRF-5, tRF-3, tiRNA-5, tiRNA-3, and i-tRF) others, such as *OncotRF*, *tRFdb*, *tRFtarget*, and *tsRFun,* present all tRFs and tsRNAs (tRF-1) but any tiRNAs, while *tRFTars* report only tRF-5s and tRF-3s (Figure 3B). On the contrary, *tsRBase* reports an extensive repertoire of different classes of tRNA-derived ncRNAs with the exception of the 5′ leader tRF, also called 5′ leader-exon tRF,^20^ which is uniquely included in *tRFUniverse*. Although both tRFUniverse and MINTbase report the tRFs from the mature tRNA detected in TCGA using MINTmap, discrepancies between these two databases were detected. The reason for these differences might be due to (i) the different versions of MINTmap used in tRFUnivese (MINTmap v.2) and MINTbase (MINTmap v.1), (ii) and different versions of small RNA-Seq data analyzed (in MINTbase were analyzed the small RNA-seq data of TCGA downloaded in 2015 while we used the 2021 release). All the different classes of tRNA-derived ncRNAs reported in *tRFUniverse* and the other databases are shown in Figure 3B.

In addition to having an extensive collection of tRNA-derived ncRNAs, one of the major strengths of *tRFUniverse* is its considerable number of implemented features and analyses. Indeed, *tRFUniverse* includes, in a single resource, most of the analyses implemented in the other resources so far (Table 1). Moreover, our web application presents several analyses, such as (i) phenotype simulation analysis; (ii) metadata-mediated correlation analyses; (iii) literature knowledge-graph analyses; (iv) dimensionality reduction; and (v) clustering analyses that are uniquely implemented in *tRFUniverse*. In Table 1, we summarized all the features and functional analyses implemented in *tRFUniverse* compared with the abovementioned databases.

**Table 1:** Comparison table of datasets, features, and analyses

	Dataset types	Interactive genome browser	Reporting aliases	Reporting targets	Target enrichment analysis	Phenotype simulation analysis	Correlation analysis	Metadata-mediated correlation analysis	Correlated gene enrichment analysis	Fragment-specific survival analysis	Literature knowledge-graph analysis	Dimensionality reduction analysis	Clustering analysis	Differential Expression Analysis	Differential survival analysis
tRFUniverse	TCGA TARGET NCI-60 Biological Fluids AGO CLASH -Seq AGO CLEAR-Seq AGO CLIP-Seq	X	X	X	X	X	X	X	X	X	X	X	X	X	X
tRFexplorer	TCGA NCI-60	X					X							X	
MINTbase	TCGA														
OncotRF	TCGA	X	X				X		X	X				X	X
tRFdb	Selected smRNA-Seq data from NCBI-SRA														
tRFtarget	–		X	X (predicted)											
tRFTar	AGO CLASH-seq AGO CLIP-Seq			X	X										
tRFTars	AGO CLASH-seq AGO CLEAR-CLIP			X											
tsRBase	Selected smRNA-Seq data from NCBI-SRA AGO CLASH-seq AGO CLEAR-CLIP			X											
tsRFun	TCGA AGO CLASH-seq AGO CLEAR-CLIP AGO CLIP-Seq			X	X					X				X	X

Table showing all the datasets, features, and analyses implemented in tRFUniverse and on the other compared databases.

Noteworthy, no other studies have ever analyzed tRNA-derived ncRNAs in pediatric tumors. Indeed, *tRFUniverse* is the first resource reporting the expression of tRNA-derived ncRNAs in all pediatric tumors available from TARGET cohorts (http://ocg.cancer.gov/programs/target). Furthermore, all the previously described functional analyses will allow users to investigate, for the first time, the involvement of tRNA-derived ncRNAs in the development of these rare pediatric malignancies.

## Discussion

tRNA-derived ncRNAs are recently attracting increasing attention from the scientific community because of their involvement in regulating gene expression through various mechanisms. Indeed, several classes of tRNA-derived ncRNAs have been found to be complex with the AGO proteins regulating gene expression at the post-transcriptional level through a miRNA-like mechanism.^34^^,^^51^^,^^52^^,^^66^^,^^67^ Moreover, several studies have linked the dysregulation of these small RNA molecules with the development and progression of several different types of human cancers, suggesting their potential clinical applications as novel diagnostic and prognostic biomarkers or as innovative molecular targets.^68^^,^^69^^,^^70^^,^^71^ However, more studies will be required to fully understand the biological functions of the tRNA-derived ncRNAs, especially in complex diseases such as cancer. In this context, bioinformatics and the increasing number of available databases and software for data analysis are providing new ways to study the biological functions of these fascinating regulatory small ncRNAs.

Since the release of the first database about tRNA-derived ncRNA in 2014,^40^ several resources have been released. However, a few focused on human cancer, such as *MINTbase*,^42^ *OncotRF*,^44^ and *tsRFun*.^49^ On the other hand, other online databases were developed to primarily provide the direct targets of the tRNA-derived ncRNAs extracted by analyzing the publicly available AGO CLASH/CLEAR/CLIP-Seq data. Examples of such databases include *tRFtarget*,^46^ *tRFTar*,^47^ *tRFTars*,^48^ and *tsRBase*.^45^ Although some of these previously mentioned resources present interesting features such as reporting molecular interactions between tRNA-derived ncRNAs and mRNAs, performing target enrichment analyses, differential expression analyses, and survival analyses, they still present some limitations that might affect their applicability in the context of cancer research. Such limitations include (i) missing tRNA-derived ncRNA classes; (ii) lacking some functional analyses; and (iii) the absence of a single integrated portal that includes most of the features already implemented in other resources in a single environment.

For this reason, we decided to implement a novel web application, named *tRFUniverse*, that includes in a single resource most of the features and analyses individually present in some of the pre-existing databases, plus other features and functional analyses that might be helpful to investigate the biological functions of the tRNA-derived ncRNAs in the context of human cancer. Moreover, *tRFUniverse* presents an easy-to-use GUI that allows users with no bioinformatics or computer programming expertise to run all the implemented analyses and easily interpret the results from interactive and downloadable tables and plots.

Noteworthy, in *tRFUniverse*, we reported, for the first time, the expression of tRNA-derived ncRNAs in all the pediatric tumor cohorts available from TARGET, providing, therefore, the first online resource capable of analyzing such molecules in these malignancies.

In conclusion, *tRFUniverse* is a user-friendly web-based application that allows users to perform several functional analyses based on tRNA-derived ncRNA expressions in TCGA, TARGET patient cohorts, NCI-60 cell lines, and human biological fluids to investigate their involvement in user-defined tumor types. To the best of our knowledge, no other online cancer-oriented resource presents, in a single environment, all the features and analyses implemented in *tRFUniverse*. Moreover, the completeness of the different classes of tRNA-derived ncRNAs reported, plus the massive amount of analyzed data, make *tRFUniverse* the most comprehensive online resource to explore tRNA-derived ncRNAs in human cancer. Our web application might be helpful to researchers in investigating the potential molecular functions of tRNA-derived ncRNAs and generating new hypotheses about their biology.

### Limitations of the study

This study presents a novel online resource for analyzing tRNA-derived ncRNAs in human cancer called tRFUniverse. Despite the extensive analysis implemented, most of their functions and mechanisms remain elusive. Therefore, the features and analyses in tRFUniverse cover only some known aspects of their biology. However, tRFUniverse also has some limitations that need to be considered. For instance, some lowly expressed tRNA-derived ncRNAs in specific tumor types may not be detected and reported by the current version of tRFUniverse. Moreover, tRFUniverse does not account for the possible post-transcriptional modifications of tRNA-derived ncRNAs, which may affect their stability, localization, and function.

## STAR★Methods

### Key resources table

**Table:** 

REAGENT or RESOURCE	SOURCE	IDENTIFIER
**Deposited data**		

tRFUniverse (source code)	GitHub	https://github.com/knowmics-lab/tRFUniverse
TCGA (small RNA-Seq)	NCBI-dbGaP	https://www.ncbi.nlm.nih.gov/projects/gap/cgi-bin/study.cgi?study_id=phs000178.v11.p8
TARGET (small RNA-Seq)	NCBI-dbGaP	https://www.cancer.gov/ccg/research/genome-sequencing/target/using-target-data/citing
NCI-60 (PRJNA390643)	NCBI-SRA	https://www.ncbi.nlm.nih.gov/sra/?term=PRJNA390643
Biological fluids (PRJNA242348)	NCBI-SRA	https://www.ncbi.nlm.nih.gov/sra/?term=PRJNA242348
Biological fluids (PRJNA415462)	NCBI-SRA	https://www.ncbi.nlm.nih.gov/sra/?term=PRJNA415462
Biological fluids (PRJNA431173)	NCBI-SRA	https://www.ncbi.nlm.nih.gov/sra/?term=PRJNA431173
Biological fluids (PRJNA445720)	NCBI-SRA	https://www.ncbi.nlm.nih.gov/sra/?term=PRJNA445720
Biological fluids (PRJNA505788)	NCBI-SRA	https://www.ncbi.nlm.nih.gov/sra/?term=PRJNA505788
Biological fluids (PRJNA527257)	NCBI-SRA	https://www.ncbi.nlm.nih.gov/sra/?term=PRJNA527257
Biological fluids (PRJNA647356)	NCBI-SRA	https://www.ncbi.nlm.nih.gov/sra/?term=PRJNA647356
AGO CLASH/CLEAR-Seq data (PRJNA217521)	NCBI-SRA	https://www.ncbi.nlm.nih.gov/sra/?term=PRJNA217521
AGO CLASH/CLEAR-Seq data (PRJNA691465)	NCBI-SRA	https://www.ncbi.nlm.nih.gov/sra/?term=PRJNA691465
AGO CLASH/CLEAR-Seq data (PRJNA296130)	NCBI-SRA	https://www.ncbi.nlm.nih.gov/sra/?term=PRJNA296130
AGO CLIP-Seq data (PRJNA129395)	NCBI-SRA	https://www.ncbi.nlm.nih.gov/sra/?term=PRJNA129395
AGO CLIP-Seq data (PRJNA176418)	NCBI-SRA	https://www.ncbi.nlm.nih.gov/sra/?term=PRJNA176418
AGO CLIP-Seq data (PRJNA248264)	NCBI-SRA	https://www.ncbi.nlm.nih.gov/sra/?term=PRJNA248264

**Software and algorithms**		

Samtools (v.1.12)	Ubuntu package	https://manpages.ubuntu.com/manpages/jammy/man1/samtools.1.html
Trim Galore (v.0.6.6)	The Babraham Institute	https://www.bioinformatics.babraham.ac.uk/projects/trim_galore/
Bowtie 2 (v.2.4.5)	Bioconda	https://anaconda.org/bioconda/bowtie2
tRFexplorer (data)	GitHub	https://github.com/knowmics-lab/tRFexplorer
MINTmap (v.2)	Jefferson	https://cm.jefferson.edu/mintmap/
Bedtools (v.2.30.0)	Bioconda	https://anaconda.org/bioconda/bedtools
Flexbar (v.2.5)	Ubuntu package	https://manpages.ubuntu.com/manpages/jammy/man1/flexbar.1.html
CLASH Analyst	GitHub	https://github.com/t50504/CLASHanalyst
RNAhybrid	Ubuntu package	https://manpages.ubuntu.com/manpages/trusty/man1/RNAhybrid.1.html
MACS2 (v.2.2.7.1)	PyPI	https://pypi.org/project/MACS2/
Laravel (v.9.19)	Composer	https://packagist.org/packages/laravel/framework
Next.js (v.12.2.5)	NPM	https://www.npmjs.com/package/next
JBrowse 2 (v.2.1.5)	NPM	https://www.npmjs.com/package/@jbrowse/react-linear-genome-view
R (v.4.2)	Ubuntu package	https://cloud.r-project.org/

### Resource availability

#### Lead contact

Further information and requests for resources and data should be directed to and will be fulfilled by the Lead Contact, Alfredo Pulvirenti (alfredo.pulvirenti@unict.it).

#### Materials availability

This study did not generate new unique reagents.

#### Data and code availability

• All data are publicly available at the accession numbers listed in the key resources table and on GitHub at https://github.com/knowmics-lab/tRFUniverse. tRFUniverse is available at https://trfuniverse.cloud/.
• The source code is available on GitHub at https://github.com/knowmics-lab/tRFUniverse.
• Any additional information required to reanalyze the data reported in this paper is available from the lead contact upon request.

### Method details

#### Resource development

The *tRFUniverse* web application has been developed using a microservice-based architecture for improved performance and availability (Figure S1). All services that make up the database are deployed in Docker containers. Container monitoring and orchestration are delegated to the Kubernetes platform.

The *tRFUniverse* web application comprises two primary services: a frontend service and a backend service. Both services employ a Kubernetes ingress service for external availability and a Kubernetes load balancer to dynamically spawn new on-demand instances based on the resource requested by the users.

The frontend service has been implemented as a stateless web app built with the Next.js framework to exploit its static site generation (SSG) and incremental static regeneration (ISR) capabilities. SSG is a technique that creates a snapshot of a dynamic web page at build time to remove the burden of computing the page for each user request. Dynamic content is automatically hydrated on request directly on the user’s browser, reducing the load on the server. ISR is a dynamic extension of SSG where the snapshot of a web page is computed only on the first user request and cached for further access by other users. These two techniques allow us to dramatically reduce the computational resources needed to provide a good user experience.

The backend service has been implemented as a stateful API (Application Programming Interface) with the Laravel framework. The backend service manages all analyses requested by the user, input validation, and coordinates access to the database containing all *tRFUniverse* data stored within a MySql database. Furthermore, the backend service uses a separate Job Service that is spawned on demand to perform the analyses a user requests. Currently, ten job services can be activated simultaneously, each managing up to three concurrent computations.

A Redis service, an in-memory key-value store database, is used for job queue management. When a user requests a new analysis, a record containing all analysis parameters is added to the Redis-based queue. Once a free job service instance is available, the first job is picked from the front of the queue and processed. Kubernetes monitors each instance continuously and spawns new ones when more resources are needed. The Redis service is also employed for caching and user session management.

Finally, communication between the frontend and the backend is achieved via REST (REpresentational State Transfer) HTTP requests and WebSocket-based real-time channels. Real-time channels are maintained by a Soketi service, a fast and resilient open-source WebSocket server.

### Quantification and statistical analysis

#### Quantification of tRNA-derived ncRNAs from public smRNA-Seq data

smRNA-Seq data in BAM format from TCGA (https://cancer.gov/tcga) and TARGET (http://ocg.cancer.gov/programs/target) cohorts were downloaded using Genomic Data Commons (GDC) Data Transfer Tool^72^ after obtaining authorization from the data access committee (DBGap Project IDs: #11332 and #73394 for TCGA; #22219 for TARGET). Subsequently, the BAM files were converted in FASTQ format using Samtools (bam2fq) (v.1.12).^73^ Instead, smRNA-Seq data for the NCI-60 (BioProject: PRJNA390643) and biological fluids (BioProject: PRJNA242348, PRJNA415462, PRJNA431173, PRJNA445720, PRJNA505788, PRJNA527257, PRJNA647356) samples were downloaded in FASTQ format from NCBI-SRA. All downloaded smRNA-Seq data in FASTQ format were quality trimmed (-q 20), and adapters were removed using Trim Galore (v.0.6.6) (https://www.bioinformatics.babraham.ac.uk/projects/trim_galore/). Trimmed reads were then mapped by Bowtie2 without allowing mismatches, insertions or deletions (-L 10 -N 0) (v.2.4.5)^74^ on a custom transcriptome generated from the tsRNA (average length 23 nts) and 5′ leader tRF (5′ leader region of the pre-tRNA - 20 nts length) sequences retrieved from Pekarsky Y et al.,^19^ and *tRFexplorer*,^21^ respectively (tRFs with post-transcriptional additions such as that of the nuclear tRNA HisGTG^75^ are not analyzed). In both cases, the tRNA sequences and their genomic coordinates used originally for making the tsRNA and 5′ leader tRF annotation were downloaded from GtRNAdb.^76^ This step allowed us to filter tsRNAs and 5′ leader tRFs from tRFs and tiRNAs since MINTmap does not analyze tRNA-derived ncRNAs generated from the pre-tRNA. After that, the reads that were not mapped to tsRNAs and 5′ leader tRFs (by Bowtie2) were then extracted in FASTQ format using the Bowtie2’s parameter “–un” and used as input for MINTmap (v.2)^77^ in order to quantify tRFs and tiRNAs (exclusive counts). On the other hand, the reads that mapped on the tsRNA\5′ leader tRF transcriptome (in SAM format) were extracted (samtools view -h -F 4), converted in BAM format (samtools view), sorted by coordinates (samtools sort), and indexed (samtools index) using Samtools (v.1.12).^73^ Subsequently, tsRNAs and 5′ leader tRFs were quantified using Bedtools (multicov) (v.2.30.0).^78^ Finally, the raw count matrices generated by MINTmap for tRFs and tiRNAs and by Bedtools for tsRNAs and 5′ leader tRFs were all merged to have a single matrix for each data project and included in *tRFUniverse*. A schematic representation of the analysis pipeline is shown in Figure S2. Only the tRNA-derived ncRNAs with a length >14nt and an average expression >0.1 Read Per Million (RPM) for each cohort were selected and included in *tRFUniverse*. Note, the impact of such a threshold might have been different in TCGA/TARGET cohorts with few samples compared to other cohorts with more samples, potentially affecting the total number of expressed tRNA-derived ncRNAs per cohort.

#### Analysis of CLASH/CLEAR-Seq data

AGO CLASH/CLEAR-Seq data (BioProject: PRJNA217521, PRJNA691465, PRJNA296130)^79^^,^^80^^,^^81^ in FASTQ format were downloaded from NCBI-SRA. Low-quality reads and adapters were removed using Trim Galore (v.0.6.6) (https://www.bioinformatics.babraham.ac.uk/projects/trim_galore/), while potential 5′ barcodes were removed using Flexbar (v.2.5).^82^ After that, the identification of the interactions between tRNA-derived ncRNAs and mRNAs was performed by the CLASH Analyst pipeline^83^ using as input (i) the processed AGO CLASH/CLEAR-Seq data (in FASTQ format), (ii) the sequences of all the expressed tRNA-derived ncRNAs included in *tRFUniverse*, (iii) and the transcripts available on Gencode (v. 40) in FASTA format. The secondary structure of binding sites of the identified interactions were finally shown using RNAhybrid^84^ as indicated in.^79^

#### Analysis of CLIP-Seq data

AGO CLIP-Seq data (BioProject: PRJNA129395, PRJNA176418, PRJNA248264) in FASTQ format were retrieved from NCBI-SRA. Replicates were merged to improve sequencing depth and analyzed by adapting the protocol published by Moore et al*.*^85^ In more detail, low-quality reads and adapters were removed using Trim Galore (v.0.6.6) (https://www.bioinformatics.babraham.ac.uk/projects/trim_galore/). After that, trimmed reads were mapped to the human genome (HG38) using Bowtie2 (v.2.4.5) (46), and then aligned reads in SAM format were converted to BAM, sorted and indexed using Samtools (v.1.12) (45). The peak calling step using MACS2 (v.2.2.7.1)^86^ was successively performed to identify the gene regions immunoprecipitated with the AGO proteins potentially containing the binding sites of the tRNA-derived ncRNAs. With this purpose, all the identified peaks were then filtered using the GenomicRanges R package^87^ in order to remove intergenic and intronic peaks and annotated following the genomic coordinates present in the GTF file downloaded from Gencode (v.40). The sequences of the filtered peaks were finally retrieved using the *getfasta* function of Bedtools (v.2.30.0).^78^ Concerning the tRNA-derived ncRNAs, the previously described pipeline used to quantify the tRNA-derived ncRNAs from the smRNA-Seq data was also used on the AGO CLIP-Seq data to identify the immunoprecipitated tRNA-derived ncRNAs. After that, their full sequences were collected and formatted in FASTA. Finally, the FASTA files containing the binding regions of the targets and the sequences of the regulators (tRNA-derived ncRNAs) were used as input for RNAhybrid^84^ to predict their interactions.

## Supplemental information

Document S1. Figures S1 and S2

Table S1. Analyzed TCGA and TARGET cohorts, related to Figure 1 Table showing the samples analyzed for each TCGA and TARGET tumor cohort.

Table S2. Analyzed small RNA-Seq data of NCI-60 cell lines, related to Figure 1 Table reporting all the small RNA-seq datasets of NCI-60 cancer cell lines analyzed for extracting tRNA-derived ncRNA expression with information about the cell type, type of tumor, and NCBI-SRA ID.

Table S3. Distribution of tRNA-derived ncRNAs expressed across analyzed datasets, related to Figure 1 Table reporting the numbers of tRNA-derived ncRNAs (for each subclass) expressed in the analyzed datasets.

Table S4. Analyzed small RNA-Seq data of human biological fluids, related to Figure 1 Table showing all the small RNA-Seq datasets of human biological fluids analyzed for extracting tRNA-derived ncRNA expression with information about the type of fluid, sequencing platform, NCBI SRA project, and run ID.

Table S5. Analyzed AGO CLASH/CLEAR/CLIP-Seq data, related to Figure 1 Table reporting all the AGO CLASH/CLEAR/CLIP-Seq data analyzed for extracting the interaction between tRNA-derived ncRNAs and other transcripts with information about data type, cell line, AGO protein, and NCI-SRA project and run ID.
