# Supplementary material for: tRFUniverse: A comprehensive resource for the interactive analyses of tRNA-derived ncRNAs in human cancer
Source: iScience. 2024 Jan 5;27(2):108810. doi: 10.1016/j.isci.2024.108810 (PMC10831894; doi:10.1016/j.isci.2024.108810)
Supplement: Document S1. Figures S1 and S2 [file mmc1.pdf]

iScience, Volume *27*

Supplemental information

tRFUniverse: A comprehensive resource for the interactive analyses of tRNA-derived ncRNAs in human cancer

Alessandro La Ferlita, Salvatore Alaimo, Giovanni Nigita, Rosario Distefano, Joal D. Beane, Philip N. Tsichlis, Alfredo Ferro, Carlo M. Croce, and Alfredo Pulvirenti

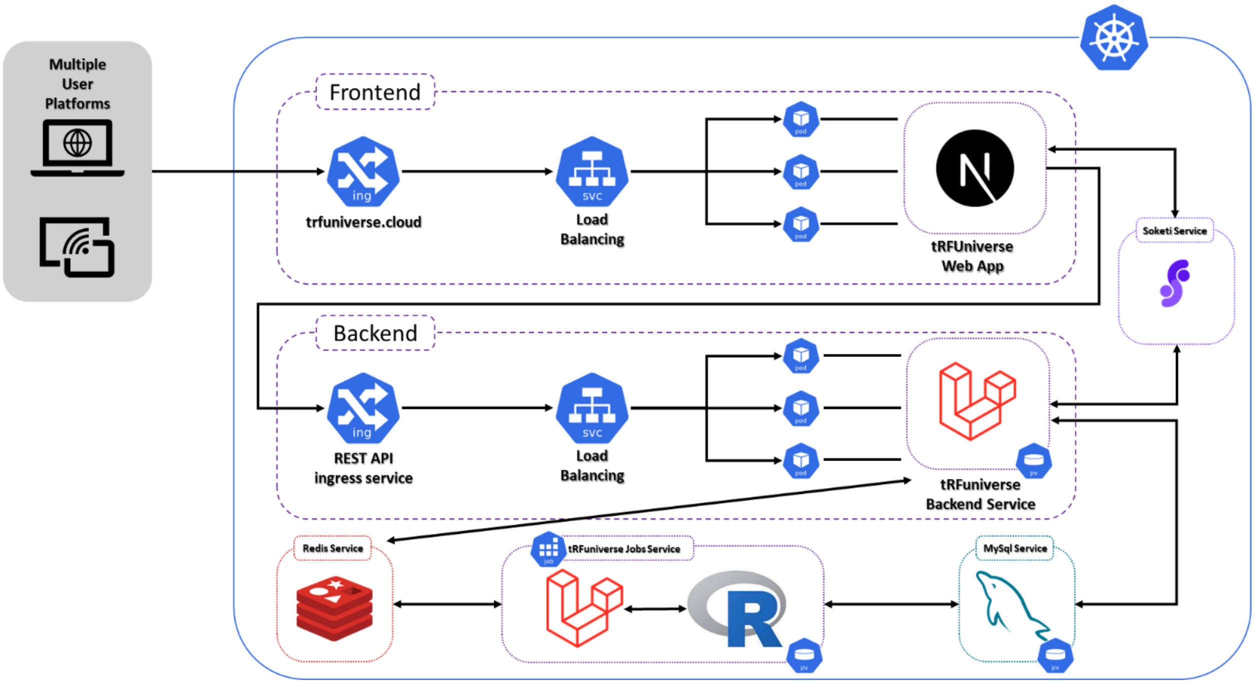

Figure S1. *Database architecture of tRFUniverse*, related to Figures 1 and 2.

Schematic representation of the frontend and backend architecture of tRFUniverse.

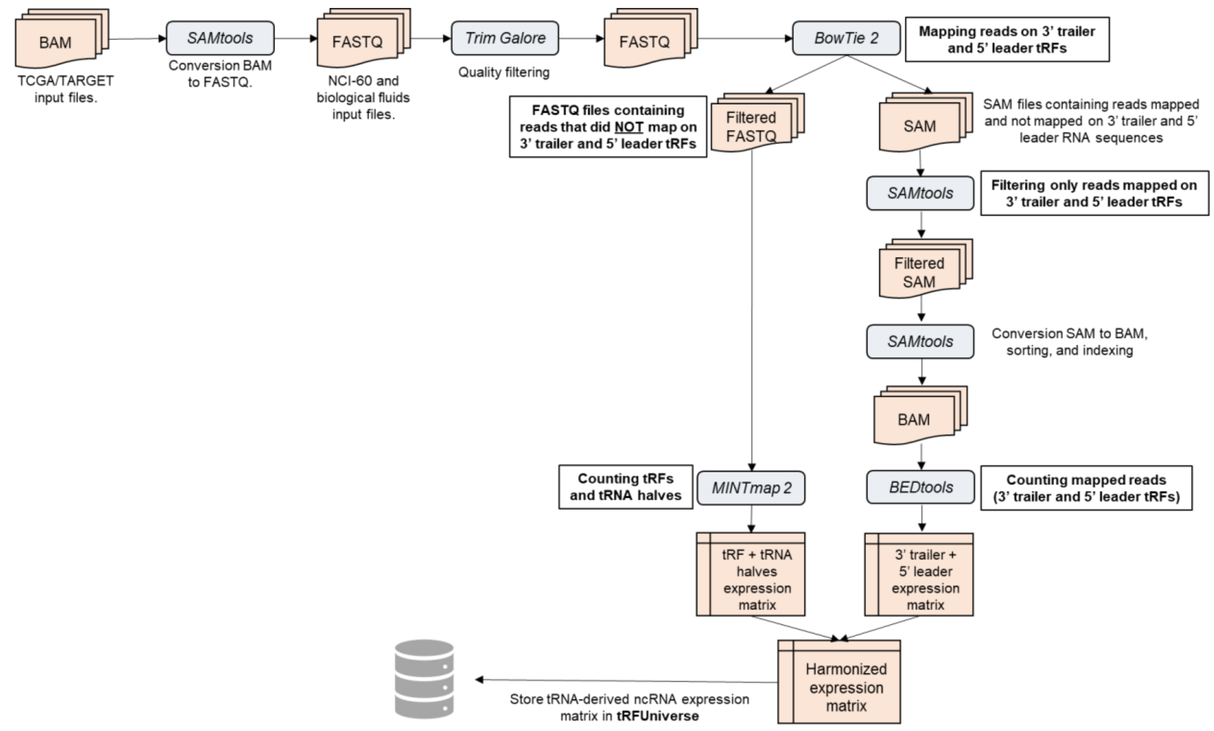

Figure S2. *tRNA-derived ncRNA pipeline for small RNA-Seq data*, related to Figures 1 and 3.

Schematic representation of the pipeline used for extracting the expression of tRNA-derived ncRNAs from the small RNA-Seq data of TCGA, TARGET, NCI-60, and human biological fluids.
